# Race/Ethnicity and Informal Caregiver Burden After Traumatic Brain Injury: A Scoping Study

**DOI:** 10.1089/heq.2020.0007

**Published:** 2020-07-08

**Authors:** Mark D. Sodders, Elizabeth Y. Killien, Lynn G. Stansbury, Monica S. Vavilala, Megan Moore

**Affiliations:** ^1^ Harborview Injury Prevention and Research Center, University of Washington, Seattle, Washington, USA.; ^2^ Department of Child, Family, and Population Health Nursing, School of Nursing, University of Washington, Seattle, Washington, USA.; ^3^ Department of Anesthesiology and Pain Medicine, School of Medicine, University of Washington, Seattle, Washington, USA.; ^4^ Division of Pediatric Critical Care Medicine, Department of Pediatrics, University of Washington, Seattle, Washington, USA.; ^5^ School of Social Work, University of Washington, Seattle, Washington, USA.

**Keywords:** traumatic brain injury, caregivers, burden, racial disparities

## Abstract

**Background:** Informal caregivers for persons with traumatic brain injury (TBI) face a range of unique issues, and racial/ethnic group differences in caregiver challenges are poorly understood. We undertook a scoping study of peer-reviewed literature to assess the quantity and quality of available research describing differences by race/ethnicity in informal caregiving roles and burden.

**Methods:** Using Arksey and O'Malley's framework and guided by the Preferred Reporting Items of Systematic Reviews and Meta-analyses Extension for Scoping Reviews, we conducted electronic searches of PubMed, CINAHL, PsycARTICLES, PsycINFO, Social Work Abstracts, Embase, and Scopus to identify peer-reviewed studies that examined TBI informal caregiver burden and reported on the influences of race or ethnicity.

**Results:** Among 4523 unique publications identified and screened, 11 studies included sufficient race/ethnicity data and were included in the analysis. Of these, six studies described civilian populations and five described military Veterans Affairs (VA). Included studies revealed that nonwhite caregivers and white caregivers use different approaches and coping strategies in their caregiving role. Some studies found differences in caregiver burden by race or ethnicity, others did not. Most were limited by a small sample size and overdependence on assessment tools not validated for the purposes or populations for which they were used. This was particularly true for race/ethnicity as a factor in TBI caregiver burden in VA groups, where essential characteristics moderate the association of race/ethnicity with socioeconomic factors.

**Conclusions:** This scoping study highlights the paucity of information on race/ethnicity as a factor in TBI caregiver burden and roles, and suggests that innovative and alternative approaches to research are needed to explore needed changes in practice.

## Introduction

Unpaid family and community caregivers of persons with traumatic brain injury (TBI) often require ongoing support in many areas for extended periods.^1–3^ Although the burden of informal caregiving is increasingly recognized,^4–7^ awareness of the problem and systems for action among health care professionals are limited.^8^ The range of issues affecting informal caregiving of patients with TBI is complex and includes the management of health information,^9–11^ behavior,^10,11^ mental and emotional health,^10,12–14^ accessing social support, assistance with life planning,^10^ and financial counseling.^10,14^ In contrast to other forms of acquired brain tissue disruption such as stroke, or Alzheimer's dementia, TBI tends to be clinically comparatively heterogeneous and to occur in younger populations with a distinct potential array of socioeconomic resources.^15–17^ Because of the variability in etiologies, risk factors, and associated medical conditions, caregiving responsibilities can differ between acquired and TBIs, which in turn can lead to differences in the burden of providing care. Currently, very little information exists on surveillance for TBI-related disability.^18^ Likewise, only limited information exists on variations in TBI-related disability by important socioeconomic factors such as race, ethnicity, or military status. However, caregiver functioning can influence outcomes after TBI, and therapies for persons who have sustained TBI can improve caregiver distress. Socioeconomic differences, including race/ethnicity and urban/rural residence, are associated with outcomes after TBI,^19–23^ including depression, anxiety, poor life satisfaction, limited access to outpatient care, worse neurocognitive performance, and higher mortality. However, it is not well described whether the burden among caregivers of persons with TBI is associated with racial/ethnic differences.^24^

To help frame community-based research on health equity aspects of home-based TBI care, we undertook a scoping study to explore the published professional literature on the association of race/ethnicity with community caregiver burden and coping mechanisms. Our hypothesis was that differences exist in measurable informal caregiver burden in the American context of race/ethnicity; a burden that may or may not be separable from socioeconomic status. Our specific aims were as follows: (1) to identify the types and quality of studies on racial/ethnic differences in caregiver burden among U.S. adult family caregivers of persons with TBI, and (2) to identify specific gaps in the professional literature related to race, ethnicity, and informal caregiving for persons with TBI. Our overall goals were to generate hypotheses and recommend avenues for future research.

## Methods

In our approach to address the study aims, we followed the methodology for scoping reviews proposed in 2005 by Arksey and O'Malley,^25^ subsequently revised and extended,^26–28^ and the Preferred Reporting Items of Systematic Reviews and Meta-Analyses (PRISMA) for Scoping Reviews.^29^ Summarized, the essential stages of a scoping study are as follows: formulating a research question; identifying potentially relevant studies; selecting and reviewing studies that address the research question; charting preselected variables of interest; and synthesizing and reporting results.^29,30^

The primary research question was: “What information is available in the current peer-reviewed medical/scientific literature on associations of race/ethnicity in the US context with TBI caregiver burden?” The secondary research questions were: (1) among the studies reviewed, what domains of caregiver assessment are represented, and (2) do significant gaps exist in the domains of caregiver assessment and, if so, what are they?

### Identification of relevant studies

Using the search terms and research strategy detailed in Appendix A1, we searched PubMed, CINAHL, PsycARTICLES, PsycINFO, Social Work Abstracts, Embase, and Scopus to identify articles related to informal/unpaid caregiving, TBI, and caregiving capacity, burden, support, or quality of life (QOL). Given that both the purpose and the methodology of this literature search were to assess the scope, that is, the breadth, of available peer-reviewed published work on our issue of concern, we set no date limits on our search query. Subject headings and keywords identified the subject areas. Boolean logic connected subjects with relevant keywords using “OR” and connected the resulting groups using “AND.”

Reference lists available electronically in systematic and other major reviews were hand-searched. All references were assembled and maintained using an embedded word-processing function (Endnote X8, Microsoft Word 2016^®^; Microsoft, Redmond, WA).

### Study selection: inclusion and exclusion criteria

**FIG. 1.**
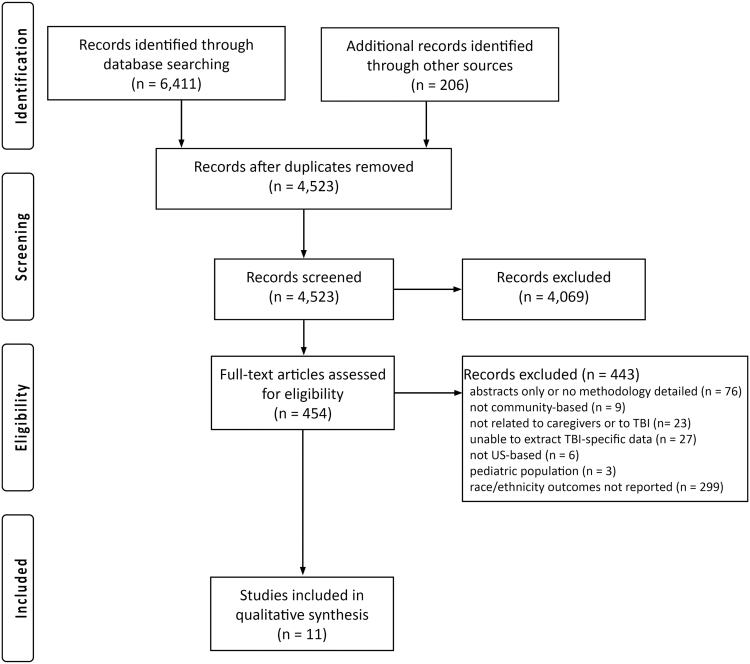
Preferred Reporting Items of Systematic Reviews and Meta-Analyses flow diagram.

Figure 1 details the search process with a PRISMA flow diagram. Included studies were as follows: (1) in English; (2) available as complete text through the University of Washington Health Sciences Library electronic access; (3) examined informal caregivers of persons with TBI; (4) examined care recipients in the postinpatient hospitalization and postinpatient rehabilitation stages of their injury recovery; (5) enrolled adult caregivers of adult care recipients (both at least 18 years old); and (4) examined any association of race or ethnicity with caregiver outcomes.

We excluded work that (1) enrolled care recipients with nontrauma brain injury (e.g., stroke, tumor, Alzheimer's dementia) or those with head injury for which TBI-specific data could not be accessed, (2) was based outside the United States, or (3) were abstracts, editorials, other commentaries, or literature reviews that did not report methodology. To avoid the confounding effects of socioeconomic/race/ethnic differences and disparities in dependent childcare, we excluded work focused on pediatric TBI.

Database searches were performed in August 2018 and updated in November 2018 and October 2019. Primary eligibility was determined by title and abstract by two authors (M.D.S., E.Y.K.). If primary eligibility was unclear, the full article was accessed and, if necessary, adjudicated independently by the senior author.

### Charting the data: data extraction and synthesis

We developed a spreadsheet (Excel^®^; Microsoft) to collate reference data and cross-check consistent analyses. Data included authors, research venue, publication year, study size; participant demographics (ages of caregivers and care recipients; race/ethnicity of caregivers and care recipients; Veterans Affairs [VA] or full military medical care recipient status); brain injury severity (usually recorded as Glasgow Coma Scale score); study type, methodology, and primary objectives; outcome measures and data collection time points; and outcomes and conclusions specifically related to race and ethnicity.

### In-depth review and synthesis of results

Two authors (M.D.S., E.Y.K.) completed a full-text review of the articles designated for secondary review and final selection. Final inclusion required some examination of caregiver burden by race and ethnicity, whether or not that examination was a primary aim of the article. Citations were clustered by caregiver assessment domains as set out in the Family Caregiver Alliance Consensus Development Conference report of 2006.^31^

## Results

Figure 1 details our search flow. Of the 4523 unique articles identified from our initial search and other articles identified from other sources, we reviewed the full text of 454 articles. Of these, 11 studies met all inclusion criteria and were included in our analysis.^32–42^

### Study designs and populations

Table 1 summarizes the characteristics of the 11 studies identified. Only three of the 11 studies that met the final inclusion criteria had as primary outcomes a potential association of TBI caregiver coping patterns and outcomes with caregiver race or ethnicity.^37,39,42^ Two other studies, in separate VA populations, included race or ethnicity as confounders or covariates.^33,38^ The remaining six studies included race or ethnicity among socioeconomic variables used in regression analyses to examine associations or predictive models.^32,34–36,40,41^ Table 2 displays the previously published instruments used to assess caregiver outcomes, of which six have been validated in informal caregivers of persons with TBI,^43–47^ and seven have been validated in care recipients with TBI but not their caregivers.^48–54^

**Table 1.** Summary of Study Characteristics in 11 Reports That Include Race/Ethnicity as a Potential Element of Caregiver Burden Among Those Caring for Persons with Traumatic Brain Injury

Study	Focus	Study design	Study group characteristics	Instruments	Results
Nabors et al.^34^	Sociodemographic predictors of caregiver burden	Cross-sectional/univariate; ANOVA; regression modeling, *p*<0.05	• Caregiver/recipient dyads: 45 • Non-VA • TBI: moderate/severe • Ages: 16–67 years	FAD; FNQ; HI-FI; interview; NON	No association between race and caregiver burden. African Americans reported less needs met compared with whites. Some differences in coping tools for African Americans vs. whites
Rivera et al.^35^	Caregiver problem-solving abilities and depression	Cross-sectional/mixed methods; principal components analysis; *p* not specified	• Caregiver/recipient dyads: 60 • Non-VA • TBI: severity unknown • Ages: 19–76 years	CBS-difficulty subscale; CES-D; PILL; SPSI-R	Race not associated with risk of depression
Sander et al.^40^	Caregiver coping strategies and subjective burden	Cross-sectional/regression modeling; *p*<0.05	• 69 primary caregivers • Non-VA • TBI: “severe closed” • Mean age: 25.8, 28.3, 45.1	GHQ; McKinlay et al. Likert scale; SSQ; WOCQ	African Americans and Hispanics had more emotion-focused coping strategies
Sander et al.^39^	Race/ethnicity and caregivers' coping	Cross-sectional/regression modeling; *p*<0.05 and 0.01	• Caregiver/recipient dyads: 195 • Non-VA • TBI: moderate/severe • Mean age: whites 48.9; nonwhites: 43.8	BSI-global severities index, anxiety subscale, depression subscale; mCAS-perceived burden subscale, caregiver satisfaction subscale, caregiver ideology subscale; WOCQ	Some differences in coping tools for nonwhite vs. white respondents, including an increased distress with more traditional caregiver ideology
Hart et al.^37^	Race, caregiving patterns, and coping	Cross-sectional; descriptive and univariate; ANOVA; *p*<0.05	• Caregiver/recipient dyads: 256 • Non-VA • TBI: moderate • Ages: 17–88 years	BSI-18; interview; SWLS	More white caregivers get formal professional treatment including Rx
Saban^41^	Quality of life in female partners of veterans with TBI	Prospective convenience sample; Pearson correlation, α 0.05	• Caregiver/recipient dyads: 40 • VA • TBI: severity not reported • Ages, mean±SD: 43.1±15.3	CRA; Ferran and Powers QLI–generic version III; PHQ-15; SF-12	Race not associated with quality of life in this sample
Phelan et al.^33^	Perceived stigma, caregiver well-being, and patient community reintegration	Cross-sectional; conventional univariate; bilinear regression, *p* unspecified	• Caregiver/recipient dyads: 564 • TBI: severity not reported • VA • Ages not reported	Devaluation of consumer families scale; everyday discrimination scale; experience of caregiving inventory; participation assessment with recombined tools—objective; interview; PROMIS depression; PROMIS anxiety; Rosenberg's self-esteem scale; UCLA loneliness scale; ZBI-SF	No association between race and caregiver-stigma by association, or felt family stigma
Winter and Moriarty^36^	Caregiver burden, satisfaction, and interpersonal functioning	Cross-sectional conventional univariate; ANOVA; bilinear regression, *p* unspecified	• Caregiver/recipient dyads: 83 • VA • TBI: mild, moderate, or severe • Ages not reported	mCAS-burden subscale and satisfaction subscale; PCRS; Williamson-winter quality of communal relationships	Hispanic ethnicity contributed to higher caregiver burden
Moriarty et al.^32^	Ecologic framework for depressive symptoms in family members of U.S. veterans with TBI	Cross-sectional secondary analysis; multiple linear regression; *p* not specified	• Caregiver/recipient dyads: 83 • VA • TBI: mild to severe • Ages: 23–67 years	CES-D SF; mCAS-burden subscale; 1 question for financial inadequacy from the REACH I study; 4 questions of social support from REACH multisite study	No association between race/ethnicity and caregiver outcomes
Moriarty et al.^38^	VA in-home program vs. standard outpatient clinic care in reducing depressive symptoms, burden, and satisfaction	Randomized-controlled trial	• Caregiver/recipient dyads: 81 • VA • TBI: mild to severe • Ages not reported	An 18-item acceptability tool; CES-D SF; mCAS-burden subscale, and caregiver relationship satisfaction subscale	Hispanic ethnicity was associated with higher caregiver burden scores
Sander et al.^42^	Association of sociocultural variables to caregivers' assessment of burden	Cross-sectional/regression modeling; *p* not specified; effect sizes reported	• Caregiver/recipient dyads: 324 • Non-VA • TBI: complicated/mild/moderate/severe • Ages reported as mean±SD • White 52.7±13.6 (18–81) • Black 48.9±14.3 (21–83) • Hispanic 50.3±14.4 (22–78)	Mayo Portland Adaptability Inventory IV; mCAS; ZBI	No difference in perceived burden between whites and Hispanics. Black caregivers reported less burden than whites. Blacks and Hispanics reported more traditional beliefs

ANOVA, analysis of variance; BSI, Brief Symptom Inventory; CBS, Caregiver Burden Scale; CES-D SF, Center for Epidemiologic Studies Depression Scale Short Form; CRA, caregiver reaction assessment; FAD, Family Assessment Device; FNQ, Family Needs Questionnaire; GHQ, General Health Questionnaire; HI-FI, head injury family interview; mCAS, Modified Caregiver Appraisal Scale; NON, Nonsupport Scale of the Personality Assessment Inventory; PCRS, Patient Competency Rating Scale; PHQ-15, Patient Health Questionnaire 15; PILL, Pennebaker Inventory for Limbic Languidness; PROMIS, Patient-Reported Outcomes Measurement Information System; QLI, Quality of Life Index; REACH I, Resources for Enhancing Alzheimer's Caregiver Health I; SD, standard deviation; SF-12, 12-Item Short Form Health Survey; SPSI-R, Social Problem-Solving Inventory-Revised; SSQ, Social Support Questionnaire; SWLS, Satisfaction with Life Scale; TBI, traumatic brain injury; UCLA, University of California, Los Angeles; VA, Veterans Affairs; WOCQ, Ways of Coping Questionnaire; ZBI, Zarit Burden Interview; ZBI-SF, Zarit Burden Interview Short Form.

**Table 2.** Validated Measures Identified

Instruments	Concepts	Domains^31^	Study
Validated in TBI caregivers			
FNQ	Importance of family needs	Skills/knowledge; potential resources	Nabors et al.^34^
HI-FI	Perceived level of burden; affective/behavioral burden; cognitive burden; physical dependency burden	Consequences	Nabors et al.^34^
mCAS-all items except mastery subscale	Caregiver ideology	Values and preferences	Sander et al.^39^
	Caregiver burden	Consequences	Sander et al.^42^
	Caregiver relationship satisfaction	Consequences	Winter et al.^32^
mCAS-burden subscale	Caregiver burden	Consequences	Moriarty et al.^38^; Winter and Moriarty^36^; Moriarty et al.^32^
mCAS-caregiver relationship satisfaction subscale	Caregiver satisfaction	Consequences	Moriarty et al.^38^; Winter and Moriarty^36^
PROMIS anxiety	Anxiety	Well-being	Phelan et al.^33^
PROMIS depression	Depression	Well-being	Phelan et al.^33^
ZBI	Caregiver strain	Well-being	Sander et al.^42^
Validated in TBI survivors, but not in caregivers of TBI survivors			
BSI-18	Depression	Well-being	Hart et al.^37^
	Anxiety		
	General severity index		
CES-D	Depression	Well-being	Rivera et al.^35^
Mayo Portland Adaptability Inventory 4	Caregiver's assessment of care recipient's status	Perception and reaction to the health and functional status of the care recipient	Sander et al.^42^
Participation Assessment with Recombined Tools-Objective	Care recipient reintegration	Specific to care recipients	Phelan et al.^33^
PCRS-interpersonal functioning subscale	Interpersonal functioning for daily activities for the patient	Consequences	Winter and Moriarty^36^
SWLS	Satisfaction with life	Well-being	Hart et al.^37^
SPSI-R	Social problem-solving abilities	Resources	Rivera et al.^35^
ZBI-SF-tested with persons with acquired brain injury and partly with persons with TBI	Caregiver strain	Consequences	Phelan et al.^33^

### Reported definitions and usage of race/ethnicity terms

Table 3 summarizes the race/ethnicity information provided in the 11 studies.

**Table 3.** Race and Ethnicity of Study Caregivers in Included Studies

Study	Study, N	Race^a^			Ethnicity^a^		
		White (%)	Black/African American (%)	Asian/other/unknown/no primary (%)	Non-Hispanic (%)	Hispanic (%)	Missing (%)
Hart et al.^37^	256	76.2	24^b^	—	—	—^b^	—
Nabors et al.^34^	45	46.7	53.3	—	—	—	—
Phelan et al.^33,c^	564	85	14	1	61.7	5.3	33
Rivera et al.^35^	60	85	11.7	—	—	3^d^	—
Saban^41^	40	72.5	27^e^	27^e^	—	27^e^	—
Sander et al.^40,f^	69	81^b^	—	—	—	—^b^	—
Sander et al.^39^	195	75	17	—	—	8^d^	—
Moriarty et al.^38,g^	81	59.3	32.1	8.7	—	12.3	—
Winter and Moriarty^36,g^	83	60.2	31.3	8.4	—	12	—
Moriarty et al.^32,g^	83	60.2	31.3	8.4	—	12	—
Sander et al.^42^	324	66.7	21.3	—	—	12^d^	—

^a^ May not add up to 100% due to rounding or missing information.

^b^ Persons of Hispanic ethnicity included in aggregate with African Americans.

^c^ Race categorized only as white, nonwhite, and unknown.

^d^ Ethnicity reported as part of race (not as an independent category).

^e^ Black, other, and Hispanic ethnicities were reported as one group in aggregate.

^f^ Only percent of total sample provided for white caregivers.

^g^ 8b and 8c collected data from the sample in 8a before randomization.

### Caregiver outcomes by race or ethnicity in non-VA populations

Building on earlier work that focused on gender as well as race/ethnicity,^40^ Sander et al. examined potential relationships between caregivers' coping mechanisms, aspects of the approach to the caregiving role, and caregiver distress.^39^ Respondents were assessed sequentially with three instruments.^47,55–57^ White and nonwhite caregivers reported using different coping mechanisms. Results were summarized as suggesting that nonwhite caregivers were more likely than white respondents to use distancing (*p*<0.01) and accepting responsibilities (*p*<0.05) as coping mechanisms, to report more traditional caregiver ideology (*p*<0.05), and that their global distress burden may be increased in association with their more traditional caregiver ideology.^39^ In a similar study 10 years later, this same group came to the opposite conclusion: use of the stated coping mechanisms appeared to decrease caregiver emotional burden among nonwhite respondents.^42^ Within both of these studies, black and Hispanic caregivers, compared with white caregivers, reported less annual income, lower educational attainment, and were more likely to be caring for a care recipient such as an extended family member other than a spouse.^39,42^

Hart et al.^37^ also examined race/ethnicity as a primary factor in caregiver outcomes. Individual interviews at baseline collected information on the relationship role of the caregiver with the care recipient, and the amount and frequency of time spent in the caregiving role. Caregivers were assessed with two published scales^58,59^; care recipients were assessed with three other published scales.^60–62^ Nonwhite caregivers reported lower educational attainment (*p*<0.01), greater likelihood of not being a spouse or parent of the recipient (*p*<0.01), lower likelihood of professional psychological support before assuming the caregiver role, and lower likelihood of receiving such support as caregivers (*p*<0.05). Nonwhite respondents were also more likely to have adopted religious community support postinjury (*p*<0.02). They spent more time daily in caregiving roles (*p*<0.001), although this appeared to be correlated with the severity of the recipient's injury. Controlled for functional abilities, African American caregivers showed equivalent life satisfaction, depression, and overall distress scores.

Nabors et al.^34^ “…(1) [assessed] the relationship of demographic characteristics of the caregiver (race, age, household income, education) to caregiver burden, family needs, family functioning and social support, and (2) [assessed] the predictors of caregiver burden as it related to affective/behavioral, physical/dependency and cognitive impairments of the person with the TBI.” Assessment instruments included four published instruments^44,63–65^ and interview questions. Study numbers were small, and missing data for two of the instruments were large (13.3%). Results were described as showing similar patterns of adjustment to TBI caregiver stressors across races, despite African American respondents reporting lower income and less access to care resources than whites. However, African American caregivers reported having less needs met compared with white caregivers.

The work of Rivera et al. focused on new-onset depression associated with informal TBI caregiving.^35^ Predictor variables included demographics and caregiver results in three functional areas, including problem-solving, caregiver burden, and caregiver health, using four published scales distinct from those used by previous researchers.^66–69^ Demographic factors, including race/ethnicity, did not affect the performance of their model.

Most of the studies in non-VA populations had small sample sizes, limiting the generalizability of their conclusions or their ability to detect smaller differences. Overall, the included articles provided conflicting information on the influence of race/ethnicity on caregiving burden.

### Caregiver outcomes and race/ethnicity in VA populations

Five of the more recent studies examined outcomes among those caring for U.S. military service veterans receiving benefits from the VA system, representing three different pools of VA benefit recipients.^32,33,36,38,41^

Saban^41^ described a pilot study specific to QOL issues for female partner/caregivers of U.S. military veterans with TBI. To identify predictors of QOL, bivariate correlations were explored. No association with race/ethnicity was identified.

Phelan et al. reported a study of 564 caregivers of VA recipients examining perceived and experienced community stigma about post-TBI and/or polytrauma, including effects on caregivers and reintegration of recipients into the community.^33^ Participants were recruited from among caregivers to veterans of active duty in Iraq or Afghanistan, discharged between September 2001 and February 2009 from four of the five Polytrauma Rehabilitation Centers located around the United States. Care intensity and amount were self-reported. A range of caregiver stressors, including perceptions of stigma, were assessed using seven additional published instruments.^70–76^ Race was not reported as a significant factor in any of the six models tested (caregiver strain, depression, anxiety, loneliness, self-esteem, caregiver/recipient reintegration; *p*=0.15–0.87).

Three studies were reported by Moriarty et al.,^32,38^ Winter and Moriarty^36^ presented data derived from the same VA-based sample population. The first, published in 2016,^38^ reported results of a clinical trial of the relative efficacy of the Veterans' In-Home program in improving family outcomes for those with TBI. Caregiver status was assessed using two of the tools previously used,^47^ and one other.^77^ Results were reported as *p*-values but without a stated significance level. This is important because 20% of the control-group dyads were reported as Hispanic origin compared with 5% of the intervention-group dyads (Fisher's exact test *p*=0.04) and, overall, the trial was reported as showing improved outcomes among intervention-group dyads.

Two subsequent reports examined additional aspects of caregiver outcomes in cross-sectional secondary analyses of this same study population. The first^36^ interviewed the previously identified participant dyads plus two additional dyads (83 total) to assess various measures of relationship quality using the methods of Winter et al.,^78^ Struchen et al.,^47^ and Lawton et al.^79^ The second approaches the previously identified study group using an “ecological framework.” Data were gleaned from baseline data collected for the original study before randomization into intervention and control groups. Measures of caregiver status were those reported for the two previous studies from this group as well as the Sun scale^80^ to assess perceived income inadequacy. As with the other VA-based studies, no associations between race/ethnicity and caregiver outcomes were found within these two studies.

### Caregiving domains assessed

**FIG. 2.**
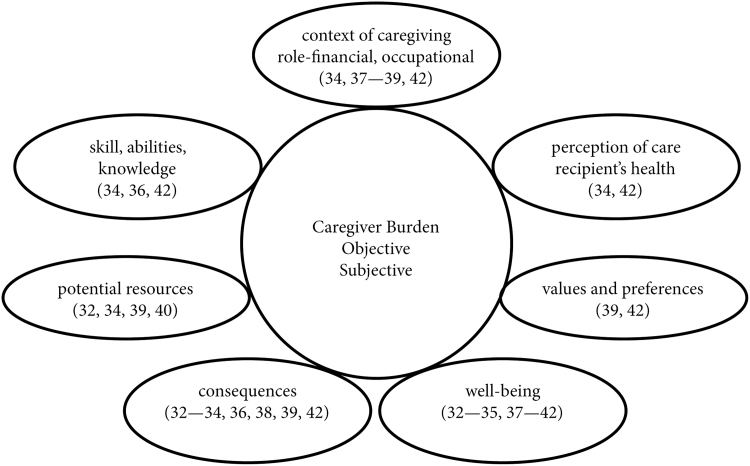
Areas of caregiver assessment based on the Family Caregiver Alliance recommendations.^31^

Among the data about race/ethnicity and caregiving burden, all seven caregiver domains^31^ were represented (Fig. 2). The most commonly measured of these was *well-being of the caregiver*. Within that domain, depression was the most commonly assessed outcome.^32–35,38^ Two studies measured the presence of symptoms of psychological distress or a psychiatric condition^37,39^ and emotional strain.^33,42^ Two studies measured anxiety.^33,34^ Single studies measured self-esteem,^33^ QOL,^41^ caregiver general health,^40^ and caregiver satisfaction with life.^37^

The next most commonly assessed domain was *consequences of caregiving*. Within that domain, the most common constructs measured were perception of burden (six studies^32,34,36,38,39,42^) and satisfaction (four studies^36,38,39,42^). Single studies assessed loneliness among caregivers,^33^ social isolation,^34^ and income inadequacy.^32^

Within the domain of *potential caregiver resources*, three studies assessed coping strategies.^34,39,40^ Single studies examined social support with a questionnaire developed for another study^32^ and perceived support needs.^34^ Within the domain of *skills, abilities, and knowledge*, single studies assessed caregiver mastery,^42^ need for medical information,^34^ and interpersonal functioning.^36^ Within the domain of *caregiver values and preferences*, two studies assessed caregiver ideology.^39,42^ Two studies assessed functioning of the care recipient by the caregiver within the domain of *caregiver's perception of health and functional status of the care recipient*.^34,42^ Five studies reported caregiving *context*,^34,37–39,42^ although these assessments were not made as specific outcomes.

## Discussion

Our systematic scoping study of English-language professional literature on the effects of race/ethnicity in the American context on informal TBI caregiver burden reveals an overall paucity of generalizable information. In the small group of studies reporting quantitative results that include race/ethnicity information, few differences were found between TBI caregiver burden as perceived by European American and non-European American groups. The few publications with such information emerged from even fewer research centers, involved relatively small study groups, focused mainly on the traditional American “black/white” constructs, and imposed statistical analyses that often appeared too advanced for the quality of the data being manipulated. Within each respective study, the domains of caregiver assessment reported showed internal logic and were well documented but, taken as a group, left important gaps. Overall, domains of caregiver assessment focused mainly on caregiver perceptions of personal well-being, potential resources available to them, and consequences of caregiving, using arrays of instruments. In contrast, only two studies, both from the same group,^39,42^ examined caregiver values and preference, both of which found important—although opposing—roles for caregiver values in decreasing caregiver burden.

All of the studies described here are remarkable for the intensity of quantitative analysis, including using quantitative analysis on responses to interview questions. Many of the instruments were not designed or validated for the specific population under study. However well-meaning, insightful, and professionally validated these instruments and their users may have been, the interposition of language and cultural assumptions in the design and deployment of such instruments must be recognized and considered.^81^ Likewise, in the work we reviewed, readers were often left to assume that considerations of sensitivity, the ability to discern differences when they exist, and specificity, the ability to recognize and reject the ascription of difference when it does not exit, were taken into consideration in analytic designs. That said, the specter of our inability to understand and communicate about—much less elicit robust, valid, reproducible quantitative data on—racial/ethnic issues in the American context haunts all of this work.

Our primary objective was to identify the types and quality of studies on racial/ethnic differences in caregiver burden among U.S. adult family caregivers of persons with TBI. However, the largest numbers of caregiver/TBI survivor dyads analyzed come from VA populations. By definition, these populations emerge from an injury-related (military) and social/economic (honorably or medically discharged from the military; registered with the VA) status. A substantial proportion of the well-documented health-related inequity in American populations is attenuated in military contexts because of equal access to and confidence in the military medical care system and the requirement for registration as “impoverished” to receive VA benefits. The interplay of these factors can defy valid or useful quantitative solutions. From a review perspective, we placed no date limits on our search, and the studies identified occur over more than two decades. The resources available and community and family relational characteristics may have changed over this time, and this may account for conflicting results. Also, informal-caregiving as a concept is broad. It is possible that work not identified in the article retrieval process exists and is appropriate to the objective of this review.

## Conclusions

Limited research is available to understand differences in how race/ethnicity affects caregiver burden in TBI. Not only is consideration of caregiver burden across multiple domains necessary for a comprehensive assessment, but studies should include diverse methods for the collection and reporting on socioeconomic data as well. Future TBI research should take care that minority groups are included appropriately, explicitly exploring the impact of race on outcomes.

## Abbreviations Used

ANOVA: analysis of variance
BSI: Brief Symptom Inventory
CBS: Caregiver Burden Scale
CES-D SF: Center for Epidemiologic Studies Depression Scale Short Form
CRA: caregiver reaction assessment
FAD: Family Assessment Device
FNQ: Family Needs Questionnaire
GHQ: General Health Questionnaire
HI-FI: head injury family interview
mCAS: Modified Caregiver Appraisal Scale
NON: Nonsupport Scale of the Personality Assessment Inventory
PCRS: Patient Competency Rating Scale
PHQ-15: Patient Health Questionnaire 15
PILL: Pennebaker Inventory for Limbic Languidness
PRISMA: Preferred Reporting Items of Systematic Reviews and Meta-analyses
PROMIS: Patient-Reported Outcomes Measurement Information System
QLI: Quality of Life Index
QOL: quality of life
REACH I: Resources for Enhancing Alzheimer's Caregiver Health I
SD: standard deviation
SF-12: 12-Item Short Form Health Survey
SPSI-R: Social Problem-Solving Inventory-Revised
SSQ: Social Support Questionnaire
SWLS: Satisfaction with Life Scale
TBI: traumatic brain injury
VA: Veterans Affairs
WOCQ: Ways of Coping Questionnaire
ZBI: Zarit Burden Interview
ZBI-SF: Zarit Burden Interview Short Form

## Appendix

### Appendix A1. Full Search Strategy for PubMed

((((((caregivers[MeSH Terms]) OR (((((Caregiving) OR caregiver) OR carer) OR care giver) OR care givers))) AND (((((Family[MeSH Terms]) OR home nursing[MeSH Terms]) OR Informal caregivers[MeSH Terms])) OR ((((((((((((((((((((((((((((((((((Family centered) OR family centered care) OR patient centered) OR patient centered care) OR home nursing) OR family) OR families) OR family member) OR family members) OR stepfamily) OR stepfamilies) OR relative) OR relatives) OR son) OR daughter) OR parent) OR parents) OR mother) OR father) OR grandmother) OR grandfather) OR grandparent) OR grandchild) OR grandson) OR granddaughter) OR friend) OR neighbor) OR lay) OR layperson) OR unpaid) OR informal) OR spouse) OR husband) OR wife))) AND ((((wounds and injuries[MeSH Terms]))) OR ((((Trauma) OR traumatic brain injury) OR tbi) OR brain injury))) AND ((((((Burden) OR capacity) OR stress) OR burnout) OR needs) OR support))
